# Escharotic Paste

**Published:** 1899-05

**Authors:** 


ESCHAROTIC PASTE. A paste consisting of equal parts of collodion and chloride of zinc is recommended as an efficient escharotic for the removal of epitheliomatous growth of the skin. It is best applied by means of a small brush.

